# Taxation without Representation

**Published:** 1888-01

**Authors:** 


### EDITORIAL DEPARTMENT.

F. E. DANIEL, M. D., EDITOR AND PUBLISHER

THIS JOURNAL, by unanimous vote of the members, was made the Official Organ of the Austin District Medical Society.

COLLABORATORS:

*Dr. R. M. Swearingen., Austin.*
*Dr. B. E. Hadra, Austin.*
*Dr. Geo. Cupples, San Antonio.*
*Dr. T. C. Osborn, Cleburne, Texas.*
*Dr. E. J. Doering, Chicago.*
*Dr. Odo Betz, Germany.*
*Dr. J. W. McLaughlin, Austin.*
*Dr. T. J. Tyner, Austin.*
*Dr. J. F. Y. Paine, Galveston.*
*Dr. E. Goldmann, San Jose, Cal.*
*Dr. H. O. Marcy, Boston.*
*Dr. E. Meierhof, New York.*

### TAXATION WITHOUT REPRESENTATION.

It is well known that it is one of the fundamental principles of this republican government that there shall be no taxation with out representation.

We no not undertake to say that the government of medical associations should be squared by that of republics, nor that the principles of the American Constitution should be the guide for the Texas State Medical Association; but there is so much equity and justice in the principles as there enunciated, that we do venture the opinion that it would be wise to adopt it in our medical congresses. We have shown on more occasions than one, that in *the workings* of the Texas State Medical Association this principle is ignored; no matter what the letter of the law may be. Our plan of electing officers, in particular, is unfair; the *majority* are not represented in the choice of officers, yet, at the same time, they are *taxed.*

This is the point to which we wish to call attention. We pointed out in our leading article last month, how the *intention* is that local societies should be represented by delegates, in proportion to their membership; and how, as only one delegate votes, (no matter how many go up from a given county society) the object is defeated. This state of affairs is made even worse by the fact that every member of every local society in affiliation with the State Association *is taxed* 50 cents per annum, whether he be a member of the State Association or not, and whether he have a voice by delegate or otherwise, or not.

Our first act at the next meeting of the State Medical Association should be to repeal the clause of the by-laws or the special act under which this tax is imposed; for, it is not only unjust, but it operates to defeat, and does, to a great extent retard, if not defeat, the very purpose for which it is declared in our constitution, the association is organized, to-wit, "for the more thorough and efficient organization of the medical profession of Texas." It keeps members away, and it prevents physicians from joining local societies. A member of the State Association pays $5 on joining, and $5 each year, as dues. Now, should his society send up a delegate supposed to represent him and four others, that delegate must collect of him and the four others $2.50, fifty cents each, and pay it into the treasury—for what? We ask, why should each member of the county societies pay 50 cents a year into the treasury of the State Association—many of them, in addition to the $5 they pay as members of that body? Have they any *quid pro quo*? any privileges or emoluments or benefits in return therefor? Not at all! On the contrary, as we have shown, they do not even have a *voice* in the choice of officers, and other matters settled by the nominating committee.

Thus, this 50 cents *per capita* on local societies represented, is a *tax on organization*—physicians must pay a tax to the State Association for the privilege of—organizing!

At the last meeting, at Austin, it will be remembered, Bell county refused to be represented, the members of the B. C. Medical Society having refused, by vote, to pay the tax.

The East Texas Medical Society, with a membership of *fifty*, refused to send delegates, because, for the privilege of doing so, they would have been taxed $25—in addition to the membership fee, and a year's dues, ($10) which would have been required of such delegates as chose to become members of the State Association; while, with our own hands, we paid for Travis County Medical Society $26—her members being supposed to be represented by the eight delegates on the ground; only one of them, however, went into the nominating convention and had anything to say as to the choice of officers. Then, we ask, why is this tax continued, seeing it has the effect of retarding, if not defeating the very purpose for which the Association came into existence? We trust one of the first things done at Galveston will be to repeal this iniquitous clause.

